# The Removal of HBV in Plasma by Extracorporeal Immunoadsorption from Plasma: A Potential Therapy of Hepatitis B Patients

**DOI:** 10.1155/2018/1269063

**Published:** 2018-12-23

**Authors:** Zhenwei Han, Xuan Lu, Yiping Tang, Yuanyuan Yang, Qiuchen Liu, Pengfei Cheng, Li Zhou, Yefu Wang

**Affiliations:** ^1^State Key Laboratory of Virology, Modern Virology Research Center, College of Life Sciences, Wuhan University, Wuhan 430072, China; ^2^Department of Laboratory, Wuhan University Hospital, Wuhan 430072, China; ^3^Animal Biosafety Level III Laboratory at the Center for Animal Experiment, Wuhan University School of Medicine, Wuhan 430071, China

## Abstract

**Objective:**

To establish a novel HBV specific immunoadsorbent for the removing of HBV particles.

**Methods:**

The anti-HBsAg monoclonal antibody was immobilized on sepharose beads to produce a sepharose anti-HBs column. Then the immunoadsorbent was evaluated and characterized by scanning electron microscopy. In addition, time-dependent effects of the eradication capacity of anti-HBsAg functionalized sepharose beads against HBV were investigated.

**Results:**

Proposed immunoadsorbents exhibited a favorable biocompatibility as well as specificity. With the optimized recycle time, the decontamination performance of HBV particles and quantity of HBsAg were assessed either by real-time quantitative PCR or ELISA, which showed that the immunoadsorbent could remove approximately 90% of the HBV and 90% of the HBsAg from human plasma samples.

**Conclusions:**

All these results indicated that the novel immunoadsorbent could effectively remove HBV particles and likely serve as a novel therapy option or at least supplementary for the treatment regimen of HBV.

## 1. Introduction

Hepatitis B virus (HBV) remains a global threat that estimated 2 billion people have been infected with worldwide. HBV chronic infected patients suffer from a high risk of liver cirrhosis and cancer that around 1 million people die from every year [1]. Current clinical trials involve modulation of the immune system and antiviral drugs include IFN-*α* and nucleoside/nucleotide analogues against HBV infection to protect liver cells [2]. However, these treatments require long-term therapy; many may have uncertain side-effects that cannot be tolerated by patients [3], especially for those with liver cirrhosis or cancer, even lead to concomitant drug resistance [4]. In recent years, most treatment strategies rely on adapting other therapeutic drugs or using combination therapies. Therefore, it is emergency to develop an additional regimen option for HBV treatment, especially for those with persistent severe HBV chronic infection. A currently discussed new treatment strategy against HBV infection aims at reducing circulating HBV particles, which are often increased in patients with HBV chronic infection and correlate with disease severity.

Immunoadsorption has been established as an effective and specific tool advantageous to plasmapheresis to remove immunoglobulin and immune complexes in cytapheresis which was used in autoimmune diseases including myasthenia gravis [5, 6], paraneoplastic neurologic syndrome [7], atopic dermatitis [8], adult immune thrombocytopenic purpura [9], low-density lipoprotein [10], systemic lupus erythematosus [5], and so on. In all these clinical cases, immunoadsorption represents a rational, effective, and relatively safe treatment option. Our aim is to establish an extracorporeal immunoadsorption system which pumps the anticoagulated blood of a patient through an extracorporeal circulation system at a specific flow rate to selectively clear away HBV pathogenic substances to achieve a therapeutic effect. In this paper we report the discovery of a new strategy of immunoaffinity column with mobilized anti-HBV surface antigen (HBsAg) monoclonal antibody on the surface of activated sepharose beads. By perfusing the column with plasma, the interaction between the HBV and the adsorbing materials clears the HBV from the patient's plasma.

## 2. Materials and Methods

### 2.1. Plasma Samples

Plasma samples of HBV infected patients and healthy people, provided from the Wuhan Blood Center (Wuhan, China), were collected and stored at – 80°C until use. All the samples were measured for DNA copy number [11] by real-time quantitative PCR and for hepatitis B surface antigen (HBsAg) protein level by ELISA method. The anti-HBsAg antibody was supplied by the Wuhan Institute of Virology at the Chinese Academy of Sciences.

### 2.2. Fabrication of Anti-HBsAg Functionalized Sepharose 6 FF Beads

Sepharose beads are a polysaccharide polymer material and have been commonly used in chromatographic separation. To activate the sepharose beads, the beads were treated with cyanogen bromide (CNBr) in a precooled alkaline potassium phosphate buffer solution for 5 min and then washed with phosphate-buffered saline (PBS), pH 8.5, resulting in activate CNBr-sepharose beads. Then immediately, the active CNBr-Sepharose beads were immersed in coupling buffer (0.1M PBS, pH 7.4) containing anti-HBsAg monoclonal antibody protein at 37°C with shaking at 120 rpm in a proper time in a rocking incubator, to obtain anti-HBsAg functionalized sepharose beads. After process of rinse, the wash-through and antibody solutions loaded were collected and dried at room temperature in a vacuum [12], followed by dilution with distilled water, which was used for calculating the coupling rate. Before process of adsorption assay, blocking buffer (0.5 M NaCl with 0.5 M ethanolamine, pH 8.3) was applied to quench the spare activate-ester group unreached on sepharose surface at 120 rpm and 37°C for 2 h in a rocking incubator. The beads were washed three times with acetate buffer (0.5 M of NaCl with 0.5 M ethanolamine, pH 4.0), Tris-HCL buffer (0.5M NaCl with 0.1 M Tris-HCl, pH 8.0), and phosphate-buffered saline (0.1M PBS, pH 7.4) successively. In control, the active CNBr-Sepharose beads bind with BSA were used to validate the nonimmunoadsorption of targets.

### 2.3. Plasma Perfusion Assays

To mimic a real immunoadsorption, anti-HBsAg functionalized sepharose beads or BSA binding sepharose (control) was loaded onto the chromatography column to generate the extracorporeal circulation system. Using a peristaltic pump to regulate the flow rate, a certain quantity of plasma at room temperature was passed over the column at a constant flow rate, and plasma samples were collected before and after adsorption.

### 2.4. Plasma Detection Assays

The samples collected through the adsorption process were measured and analyzed for HBsAg, anti-HBs, HBeAg, anti-HBe, and HBcAb protein levels and HBV DNA copy number by ELISA and real-time quantitative PCR, respectively.

### 2.5. Data Analysis

When appropriate, data were obtained from at least three independent experiments and expressed as mean ± SD. For comparison of the mean of two groups, the statistical significance was measured by Student's* t*-test. To compare the difference between multiple groups, statistical significance was analyzed using a one-way analysis of variance (ANOVA) followed by Newman-Keul's test. Calculations were performed with GraphPad Prism Statistical Software (GraphPad Software Inc., San Diego, CA) according to the methods of our previously paper [13]. Statistical significance was defined as* P* < 0.05 or* P *< 0.01.

## 3. Results

### 3.1. Fabrication of HBV Specific Sepharose Beads

**Figure 1:**
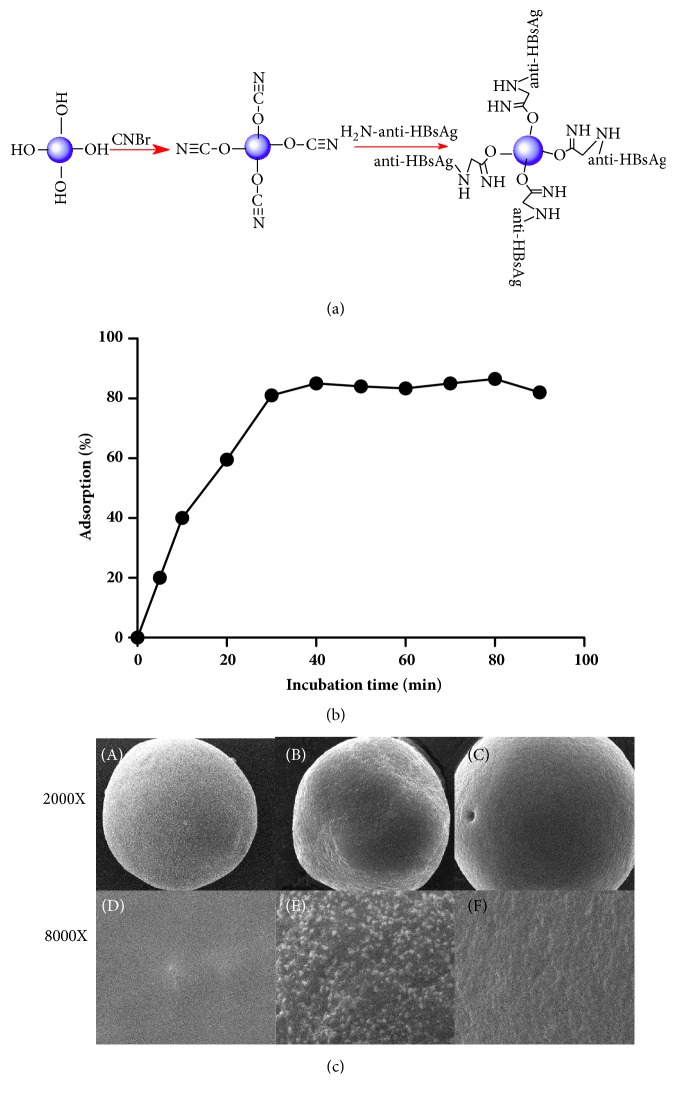
**Sepharose 6 FF was activated by CNBr through their hydroxyl groups (-OH)**. (a) Schematic representation of anti-HBsAg functionalized sepharose 6 FF. (b) Time-point optimization of the incubation conditions. 30 min as the optimal time for maximal absorption. Shown is representative of at least 3 individual experiments. (c) Scanning electron micrograph (SEM) images showing surface morphology. (A) (2000X) and (D) (8000X) showed bare CNBr-functionalized sepharose 6 FF beads; (B) (2000X) and (E) (8000X) showed anti-HBsAg functionalized sepharose 6 FF beads; and (C) (2000X) and (F) (8000X) showed anti-HBsAg functionalized sepharose 6 FF beads adsorbed with plasma.

The sepharose beads have been applied frequently to synthesis of immunoadsorbents for plasmapheresis therapy, owing to the stabilization in chemical characters and structure. In present study, active CNBr-functionalized sepharose beads [5], high cross-linked spherical sepharose, was adopted as support matrixes. Schematic representation of preparation of CNBr-functionalized sepharose 6 FF beads are shown in Figure 1(a). As the anti-HBsAg protein was immobilized covalently to attach HBV particles, the coupling incubation time for anti-HBsAg monoclonal antibody attaching to sepharose 6 FF beads was optimized. The results showed that the adsorption rate has increased from 0 min to 30 min. During the incubation time more than 30 min, the adsorption rate did not have significant change. So we considered that 30 min for incubation was the optimum time (Figure 1(b)).

Scanning electron micrograph (SEM) images showed the surface structure of active CNBr-functionalized sepharose 6 FF beads and anti-HBsAg functionalized sepharose 6 FF beads with or without plasma absorption. They were depicted, respectively, in Figure 1(c). Obviously, significant morphological changes rose after anti-HBsAg coupling. Bare active CNBr-functionalized sepharose 6 FF beads possess a glossy and smooth surface structure, while numerous raised granules were observed on the surface of affinity adsorbents. Compared with the unabsorption anti-HBsAg functionalized sepharose 6 FF beads, the affinity adsorbents exhibit coarser surface containing many bulbiform small bulges. So we hypothesized that anti-HBsAg had been immobilized successfully onto the substrate surfaces (Figure 1(c)).

### 3.2. Selectivity and Biocompatibility of Anti-HBsAg Functionalized Adsorbents

The blood compositions are import for the body, which keeps the balance of homeostatic. Nonspecific adsorption of anti-HBsAg functionalized adsorbents had been investigated which was a major concern of all extracorporeal immunoadsorbents since that great losses of useful and essential components in blood samples resulted in a troublesome issue relating to the safety that was unacceptable. Herein, effects of adsorbents on biochemical components were assessed using a batch adsorption system and examined under aseptic condition simulating clinically operations. The results are all analyzed and summarized in Table 1. It was assumed that innocent eliminations of normal blood components were inevitable in immunoadsorption process. As shown in Table 1, contents of AST, GLU, CPK, TP, and TC which represent components in plasma samples were not significantly changed (p>0.05), which demonstrated nonspecific adsorption is controllable and avoidable for the anti-HBsAg functionalized adsorbents. Other components like UREA, Crea, ALB, or VB12 were decreased significantly seemingly after adsorption assay by analysis of paired students'* T*-test, whereas the retention amounts which were somewhat negligible for the final contents were still within the allowable ranges of normal value for each individuals as indicated.

### 3.3. Recycle Optimization for HBV Affinity Adsorption

**Figure 2:**
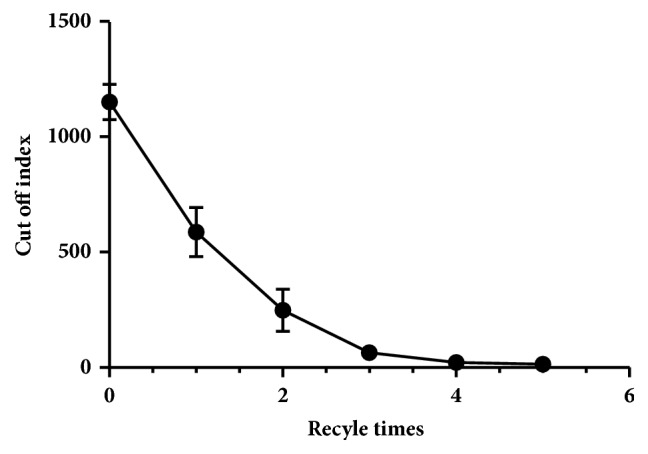
**Recycle optimization for HBV affinity adsorption**. The affinity capacity of HBV by the immunoadsorbent was detected at recycle time(s) from 1 to 5.

Time tissue of blood circulation is the key concern relative with the safety and efficiency during the extracorporeal therapy process using immunoadsorbents. Recycling of the immunoadsorbent would be an optimal scheme for the use of the activated materials. The results in Figure 2 demonstrated that recycle for 3 times of affinity adsorption could cause more than 90% eradication of HBV in plasma.

### 3.4. Comparation of the Adsorption Rates by Detection of HBsAg Levels and HBV DNA

**Figure 3:**
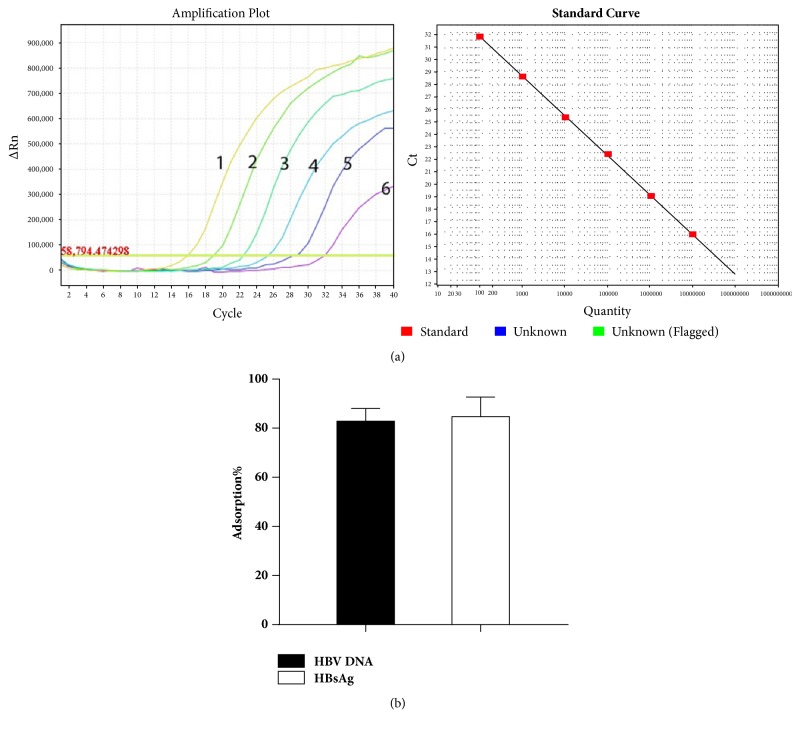
**Comparation of the adsorption rates by detection of HBV DNA copies and HBsAg levels**. (a) Establishment of real-time PCR assay for HBV detection. Standard curves for real-time PCR assays. Standard plasmids ranging from 1×10^3^ to 1×10^9^ copies/ml were run in real-time quantitative PCR mixtures to generate standard curves. R value = 0.9953. (b) The adsorption rates for HBV DNA copies and HBsAg were determined and compared (n=4).

HBV Dane particles are filamentous and spherical bodies containing HBsAg, but these filamentous and spherical bodies lack DNA. Thus, the adsorption rates of HBsAg and HBV DNA are different. In this study, we established a real-time quantitative PCR assay for HBV DNA detection. As shown in Figure 3, there were no significant discrepancies of adsorption efficiency by detection of either HBV DNA or HBsAg.

### 3.5. Batch Adsorption of HBV Particles from HBV Patients

**Figure 4:**
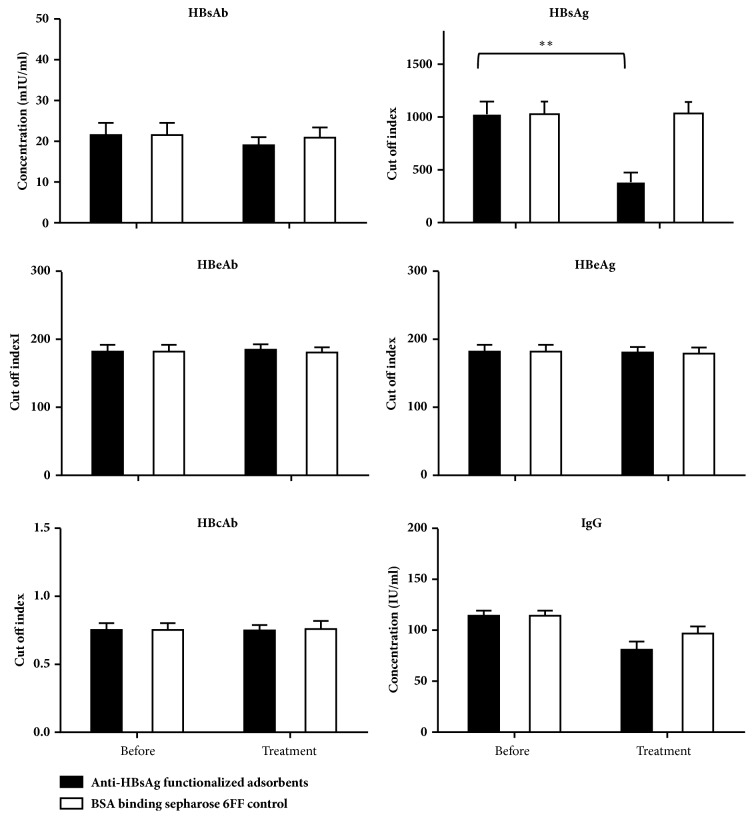
Changes of HBsAg and HBV relative protein in plasma through the anti-HBsAg functionalized adsorbents.

For the plasma perfusion, the plasma samples were collected and the HBsAg, anti-HBs, anti-HBe, HBeAg, HBcAg, and immunoglobulin (Ig) G levels were measured and compared. The presence of HBsAg indicates HBV titer in plasma. The levels of HBsAg in plasma before and after anti-HBsAg functionalized adsorbents treatment could be used to calculate the adsorption rates. In this study, the HBsAg was adsorbed well by the Sepharose 6 FF-anti-HBsAg, while there were no significant changes in the levels of anti-HBs, anti-HBe, HBeAg, HBcAg, or IgG (Figure 4). The results showed that the Sepharose 6 FF-anti-HBsAg functionalized adsorbents can capture the HBV particles with the high specificity.

## 4. Discussion and Conclusion

The current study had established a novel HBV specific immunoadsorbents, which could be combined with blood purification technique. In this study, HBV surface protein specific monoclonal antibody (anti-HBsAg) had been immobilized onto CNBr-functionalized sepharose 6 FF beads. This antibody could form a covalent bond through cyanogen bromination binding to the sepharose 6 FF by cyanate esters and then binds the HBsAg to clear the HBV virion through antibody-antigen specific adsorption. Subsequently, anti-HBsAg functionalized adsorbents have been determined available and efficiency for eliminate of HBV particles by detection both HBsAg and HBV DNA. The biocompatibility and specificity were confirmed using batch adsorption process spontaneously. Meanwhile recycle optimization for anti-HBsAg functionalized adsorbents usage was performed which indicated that recycle for 3 times of affinity adsorption could cause more than 90% eradication of HBV in plasma.

In summary, anti-HBsAg functionalized adsorbents introduced in this work exhibit pretty well potential for HBV removal and this approach could establish a novel therapy option or at least as a combination supplementary therapy strategy with antiviral drugs for the treatment regimen of HBV.

## Figures and Tables

**Table 1:** Changes of blood components after treatment (n=10).

Blood biochemical indexes	Untreated	Treated	Reference range	P value
AST (U/L)	31.23 ± 3.223	29.84 ± 3.453	0 - 45	NS
GLU (mmol/L)	4.35 ± 0.20	3.44 ± 0.32	3.61-6.11	NS
UREA (mmol/L)	4.33 ± 0.3872	3.53 ± 0.3982	1.8-7.1	*∗*
CPK (U/L)	121.34 ± 12.32	119.32 ± 12.32	25-170	NS
TP (g/L)	61.57 ± 1.44	57.34 ± 1.60	60.0-78.0	NS
Crea (*μ*mol/L)	46.34 ± 1.18	43.48 ± 1.48	59.00-104.00	*∗*
TC (mmol/L)	3.416 ± 0.83	3.174 ± 0.78	<5.30	NS
ALB (g/L)	36.28 ± 2.79	34.2 ± 2.50	34.0-48.0	*∗∗*
VB12 (pmol/L)	297.9 ± 3.12	267.32 ± 2.89.3	141.00-698.00	*∗*

AST, aspartate aminotransferase; GLU, blood glucose; UREA, urea nitrogen; CPK, creatine phosphate kinase; Crea, creatinine; TP, total protein; TC, total cholesterol; ALB, albumin; paired T-test analysis was used. Results were shown as mean ± SEM. NS, not significant. *∗*, 0.01 < p < 0.05;*∗∗*, p < 0.01.

## Data Availability

The data used to support the findings of this study are available from the corresponding author upon request.
